# Health education needs of pregnant women regarding newborn inborn errors of metabolism based on a social ecological model: a qualitative study

**DOI:** 10.3389/fpubh.2026.1867051

**Published:** 2026-09-01

**Authors:** Hao Yang, Fenghua He, Dan Xia, Xihong Wang, Yujia Guan, Hongqian Liu

**Affiliations:** 1 Department of Center for Medical Genetics, West China Second University Hospital, Sichuan University, Chengdu, China; 2 Key Laboratory of Birth Defects and Related Diseases of Women and Children (Sichuan University), Ministry of Education, Chengdu, China; 3 West China School of Public Health and West China Fourth Hospital, Sichuan University, Chengdu, Sichuan, China; 4 Department of Pediatric Urinary Disease Center Nursing, West China Second University Hospital, Sichuan University, Chengdu, Sichuan, China; 5 WCSUH-Tianfu·Sichuan Provincial Children's Hospital (Sichuan Provincial Children's Medical Center), Chengdu, Sichuan, China

**Keywords:** health education needs, inborn errors of metabolism, newborn screening, pregnant women, qualitative research, social ecological model

## Abstract

**Background:**

This study explored pregnant women's perceptions, experiences, and health education needs regarding newborn inborn errors of metabolism (IEM) and newborn screening, and examined how these needs operate across individual, interpersonal/healthcare-organizational, and community/policy-environment levels.

**Methods:**

A descriptive phenomenological qualitative design was used. Fifteen pregnant women (N1–N15) were purposively recruited from one national regional medical center between October 2025 and January 2026 for one-to-one, face-to-face, semi-structured interviews lasting 30–45 min. Two trained researchers conducted the interviews. Recordings were transcribed verbatim and managed in *NVivo*. Two researchers independently coded the data; unresolved disagreements were adjudicated by a third researcher. Data were analyzed using Colaizzi's seven-step method, and inductively generated themes were subsequently organized using a three-level consolidation of the social ecological model. All participants received their transcripts for verification, and the final three interviews confirmed thematic saturation.

**Results:**

Three overarching analytic levels and 16 subthemes were identified. Individual-level needs concerned conceptual boundaries, screening value, personalized risk information, graded management of abnormal results, uncertainty-related psychological support, and probability communication. Interpersonal and healthcare-organization needs concerned time-linked flowcharts, result notification, operational checklists, repeat-testing/referral navigation, and family communication with privacy protection. Community and policy-environment needs concerned post-diagnosis support, affordability and assistance, authoritative information access, staged reminders, and understandable multimodal delivery. Participants' accounts showed that their needs extended beyond knowledge acquisition to actionable, time-sensitive pathways linking antenatal education with postnatal screening and follow-up.

**Conclusions:**

Pregnant women's health education needs regarding newborn IEM are multi-level, time-sensitive, and action oriented. Interventions should combine plain-language risk communication and coping support at the individual level, family-inclusive communication and closed-loop service processes at the interpersonal/organizational level, and authoritative information, financial navigation, and accessible multimodal support at the community/policy level.

## 1 Background

Inborn errors of metabolism (IEM) are mostly monogenic disorders. Although the incidence of each individual condition is low, their cumulative burden is substantial. Clinically, affected neonates often appear normal at birth, the onset is frequently insidious, and progression can be rapid. Once a metabolic crisis occurs, irreversible neurological injury or even death may follow (1). With advances in diagnostic techniques and follow-up systems, increasing evidence indicates that achieving early detection, early diagnosis, and early treatment for treatable IEM can substantially improve long-term outcomes and reduce family and societal burden (2, 3). Therefore, developing health education and screening systems that enable identification and intervention before symptom onset is an important component of maternal and child health and birth-defect prevention (4).

Newborn screening (NBS) is one of the key public health strategies for achieving this goal. Since Guthrie proposed a phenylketonuria screening method based on dried blood spots (5), the selection and evaluation of screening items have long followed the Wilson-Jungner principles for population screening (6). The application of tandem mass spectrometry (MS/MS) has enabled a single test to cover multiple amino acid, organic acid, and fatty acid oxidation disorders, thereby driving the rapid expansion of screening panels (7). At the same time, policy frameworks such as the US initiative toward a uniform screening panel and system have provided useful references for program standardization (8). Although the scope and composition of NBS programmes vary across regions worldwide, the overall trend is continued expansion (9). In China, newborn screening began in the 1980s and has gradually evolved into a relatively systematic management and technical framework (10). In some regions, expanded screening by MS/MS and genetic testing has been used to refine the disease spectrum and genetic characteristics of IEM (11), and studies of newborn screening with targeted sequencing (NESTS) suggest that genetic approaches may improve detection and confirmatory efficiency (12, 13).

Despite continuing advances in screening technology and policy systems, the benefits of NBS still depend heavily on families' understanding of screening purposes, procedures, repeat testing after positive results, and diagnostic pathways. Questionnaire studies in China have shown that parental awareness and understanding of newborn screening remain insufficient, information sources are fragmented, and perceptions of screening significance, result interpretation, and subsequent management are uneven (14). During screening service delivery, healthcare professionals-especially those responsible for perinatal care and health education-often act as key gatekeepers of information for pregnant women and families, and the timing, format, and comprehensibility of information provision influence the quality of informed decision-making (15). Increasingly, health education is being moved forward into pregnancy. Randomized studies suggest that systematic prenatal education can improve parental knowledge and attitudes toward NBS and residual dried blood spot management (16), and health education interventions among pregnant women have also been shown to improve awareness and acceptance of specific screening programmes (17, 18).

At the same time, the unintended consequences of screening highlight the importance of health education and communication quality. In the context of expanded screening, false-positive results may increase parental anxiety, distort perceptions of child vulnerability, and heighten family stress (19–22). False-positive findings may also lead to increased short-term healthcare utilization and unnecessary medical visits (23). Therefore, international recommendations on NBS communication emphasize the need to explain screening purposes, the meaning of results, and repeat-testing procedures in plain, clear, and actionable language, while providing emotional support and risk communication at key time points (24).

In China, newborn screening programmes have developed over many years and achieved substantial progress, yet new challenges remain in expanded screening, genetic counseling, follow-up management, and public health education. With the introduction of next-generation sequencing and related technologies, exploratory targeted-sequencing-based screening has shown promise, but it has also increased both the complexity of screening information and the demand for informed communication. Meanwhile, surveys of prospective parents and parents indicate persistent gaps in awareness and information access regarding newborn screening, underscoring the need to move health education into pregnancy and to attend to the comprehensibility, accessibility, and actionability of educational content (25). Previous studies have also suggested that newborn screening-especially expanded screening-may be accompanied by false-positive or indeterminate results, which can provoke anxiety, stress, concerns about child vulnerability, and subsequent effects on healthcare utilization and parent-child interaction; improved communication and education are considered key strategies for mitigating these psychosocial impacts. Relevant guidelines and studies emphasize improving the clarity, timing, and consistency of communication about newborn screening and exploring systematic prenatal education and informed decision support. Health literacy is regarded as a core capability affecting how people access, understand, and use health information, and it provides an important lens for designing educational content and delivery formats during pregnancy (26).

In health-promotion research, social ecological models conceptualize health behavior as the product of influences operating at individual, interpersonal, organizational, community, and policy levels (27, 28). For this study, McLeroy's model (27) was used as the operational framework because it directly links health-promotion interventions to these levels. To preserve the inductive structure of the data while avoiding an overextended five-part presentation, the five levels were transparently consolidated into three analytic domains: individual; interpersonal and healthcare organization; and community and policy environment. This consolidation recognizes that family communication and healthcare-service processes are analytically distinct but interact closely during pregnancy and newborn screening. Health literacy-including the ability to access, understand, appraise, and use information-provides a complementary lens for explaining how people translate information into decisions and actions (26, 29).

Current research on pregnant women's health education needs related to newborn IEM and screening has been dominated by quantitative surveys, which are limited in their ability to capture women's deeper experiences and preferences regarding information content, modes of presentation, channels of access, timing of communication, and emotional support in real-life contexts. Phenomenological qualitative research is well suited to exploring how individuals construct meaning from lived experience and is therefore appropriate for assessing health education needs and identifying core intervention elements (30).

Against this background, this study used a descriptive phenomenological approach guided by a social ecological model to explore pregnant women's experiences and needs regarding health education on newborn IEM. It aimed to identify needs across individual, interpersonal/healthcare-organizational, and community/policy-environment levels and to generate evidence for staged, feasible, and evaluable antenatal education linked to postnatal screening and follow-up.

## 2 Methods

### 2.1 Study design

This study used a descriptive phenomenological design guided by McLeroy's social ecological model (27). The phenomenological component was used to explore participants' lived experiences and meanings, whereas the social ecological model was applied only after inductive theme development as an interpretive and organizational framework. Interview data were analyzed using Colaizzi's seven-step method (31).

### 2.2 Participants and sampling

Participants were pregnant women attending or being followed up in obstetric outpatient clinics, genetic counseling clinics, or maternal healthcare services at a national regional medical center in China. Recruitment and interviews occurred from October 2025 to January 2026. Purposive sampling sought variation in trimester, mode of conception, education, residence, parity, socioeconomic circumstances, and relevant reproductive or family history. Fifteen eligible women were approached; all agreed to participate, none declined, and none withdrew. No financial or material compensation was provided. Sample adequacy was judged through information richness and thematic saturation (32). Themes were stable by interview 15, and interviews N13–N15 produced no new subthemes.

### 2.3 Eligibility criteria

The inclusion criteria were as follows: (1) pregnancy confirmed by prenatal ultrasonography; (2) primary-school education or above, normal language communication ability, and the ability to clearly express personal experiences; and (3) willingness to participate, provide written informed consent, and agree to audio recording.

The exclusion criteria were: (1) a history of severe psychiatric illness, marked communication barriers, or cognitive/mental status judged unsuitable for interview; (2) obvious discomfort during the interview preventing completion; (3) severe complications of other systems or critical illness; and (4) substantial missing key information that could not be supplemented.

### 2.4 Interview guide and data collection

Semi-structured interviews were used. The interview guide was informed by the literature, the social ecological model, research-team discussion, and expert consultation, following principles for developing semi-structured interview guides (33). Two pilot interviews were used to refine wording, sequencing, and probes before the final guide was applied. The guide addressed gestational and contextual concerns, understanding of newborn IEM and newborn screening (NBS), information sources and credibility, risk perception, barriers to obtaining information and completing follow-up, priority educational content, preferred timing and formats, and features of an ideal education programme. Between October 2025 and January 2026, interviews were conducted by two researchers: one male doctorally prepared nurse and one female master's-prepared nurse. Both were clinical staff at the study hospital and had completed systematic training in qualitative research, communication, and interviewing. Neither interviewer had a prior personal or therapeutic relationship with the participants or had provided their clinical care. During consent, participants were informed that the interviewers were hospital nurse researchers and were told the study purpose; no analytic hypotheses were disclosed. Face-to-face interviews were conducted in private reception rooms, offices, or meeting rooms. No family members or other healthcare professionals were present. Interviews lasted 30–45 min, were audio-recorded, and were supported by field observations and reflexive notes documenting pauses, emotional changes, and emphasized content. Interviewers used open questions and neutral probes, avoided leading language, and remained alert to how their clinical backgrounds might influence questioning and interpretation. No repeat interviews were conducted. Recordings were transcribed verbatim within 24 h, and the complete transcripts were returned to all participants for verification.

### 2.5 Data analysis

Interview recordings were transcribed verbatim within 24 h, checked against the audio files and field notes, and managed using *NVivo* qualitative data-analysis software. Data collection and analysis proceeded concurrently. Two researchers with nursing backgrounds independently read and coded every transcript. Following Colaizzi's method (31), they: (1) read the complete transcripts repeatedly; (2) extracted significant statements; (3) formulated meanings and initial codes while retaining the original context; (4) compared codes and clustered related codes into candidate subthemes; (5) integrated subthemes into comprehensive thematic descriptions; (6) refined the fundamental structure and boundaries of each theme; and (7) returned to the transcripts and participant-verified text to confirm fit. The two coders compared their independently developed code sets and maintained an audit trail of coding decisions. Disagreements were first discussed; any disagreement that remained unresolved was adjudicated by a third researcher. Themes were developed inductively before being mapped to the social ecological framework, thereby reducing the risk that the framework predetermined the findings.

### 2.6 Application of the social ecological model

After data-driven inductive theme generation, McLeroy's individual, interpersonal, organizational, community, and policy levels (27) were consolidated into three reporting domains: (1) individual level, comprising cognition, risk understanding, emotional coping, and actionable skills; (2) interpersonal and healthcare-organization level, comprising family support, clinician-patient communication, and closed-loop service processes; and (3) community and policy-environment level, comprising social-support resources, affordability and financial protection, authoritative information environments, privacy, and institutional support. This was a pragmatic analytic consolidation rather than an assertion that interpersonal and organizational processes are theoretically identical.

### 2.7 Rigor and quality control

Several strategies strengthened credibility, dependability, confirmability, and transferability. First, independent double coding, an audit trail, team discussion, and third-researcher adjudication were used to challenge premature interpretations. Second, the developing themes were repeatedly checked against verbatim transcripts, including disconfirming or less common accounts. Third, complete transcripts were returned to all participants for verification. Fourth, interviews N13–N15 were used to test thematic saturation and produced no new subthemes. Finally, reporting was checked against the Consolidated Criteria for Reporting Qualitative Research (COREQ) (34).

### 2.8 Ethical considerations

This study was approved by the Ethics Committee of West China Second University Hospital, Sichuan University (Medical Research 2023, Ethics Approval No. 104). Informed consent was required and obtained from every participant before interview and audio recording. Participants received information about the study purpose, procedures, potential benefits and risks, confidentiality, voluntary participation, and their right to pause or withdraw without affecting care. Data were de-identified and stored on password-protected computers accessible only to the research team.

## 3 Results

### 3.1 Participant characteristics

Fifteen pregnant women (N1–N15) completed the face-to-face interviews; no participant declined or withdrew. Mean age was 31.5 ± 6.4 years (range 20–41). Five women were in the first trimester, six in the second, and four in the third. Ten conceived spontaneously and five through *in vitro* fertilization (IVF). Nine were nulliparous and six had given birth previously. Eleven lived in urban areas and four in rural areas. Educational attainment ranged from primary school to doctoral level: three had junior-high education or below, two had senior-high/secondary-vocational education, one had junior-college education, four had bachelor's degrees, three had master's degrees, and two had doctoral degrees. Annual family income ranged from below RMB 50,000 to above RMB 200,000. Three reported a history of genetic disease, five a history of adverse miscarriage, and six a relevant family history. Details shown in Table 1.

**Table 1:** Demographic, obstetric, socioeconomic, and relevant history characteristics of participants.

Panel A. Demographic and obstetric characteristics					
ID	Age	Gestational age	Gravidity and parity	Mode of conception		Residence
N1	27	14 + 3	G3P0	IVF		Urban
N2	33	10 + 4	G1P0	Spontaneous conception		Urban
N3	21	22 + 3	G2P0	Spontaneous conception		Urban
N4	27	10 + 3	G3P1	Spontaneous conception		Urban
N5	37	12 + 3	G3P0	IVF		Urban
N6	31	13 + 1	G1P0	Spontaneous conception		Urban
N7	35	14 + 3	G2P1	Spontaneous conception		Urban
N8	36	21 + 2	G3P1	Spontaneous conception		Rural
N9	25	9 + 5	G1P0	IVF		Urban
N10	41	24 + 3	G3P2	Spontaneous conception		Rural
N11	20	29 + 2	G1P0	Spontaneous conception		Rural
N12	40	31 + 3	G5P1	IVF		Urban
N13	36	33 + 1	G3P0	Spontaneous conception		Urban
N14	32	28 + 4	G3P1	Spontaneous conception		Urban
N15	32	19 + 1	G4P0	IVF		Rural
**Panel B. Socioeconomic and relevant history characteristics**						
**ID**	**Education**	**Occupation**	**Yearly family income**	**History of genetic disease**	**History of adverse miscarriage**	**Family history**
N1	Bachelor's degree	Sales employee	RMB 150,000–200,000	Yes	Yes	Yes
N2	Doctoral degree	Healthcare worker	RMB >200,000	Yes	No	Yes
N3	Bachelor's degree	Civil servant	RMB 100,000–150,000	No	No	Yes
N4	Bachelor's degree	Company employee	RMB 100,000–150,000	No	No	No
N5	Master's degree	Manager	RMB 150,000–200,000	Yes	Yes	Yes
N6	Junior college	Self-employed	RMB 50,000–100,000	No	No	No
N7	Master's degree	Teacher	RMB 150,000–200,000	No	No	No
N8	Junior high school	Farmer	RMB < 50,000	No	No	No
N9	Bachelor's degree	Self-employed	RMB 100,000–150,000	No	No	No
N10	Primary school	Farmer	RMB < 50,000	No	No	No
N11	High school	Self-employed	RMB 50,000–100,000	No	No	No
N12	Secondary vocational school	Accountant	RMB 100,000–150,000	No	Yes	Yes
N13	Doctoral degree	Teacher	RMB >200,000	No	Yes	Yes
N14	Master's degree	Technical staff	RMB >200,000	No	No	No
N15	Junior high school	Farmer	RMB < 50,000	No	Yes	No

GA, gestational age; G, gravidity; P, parity; IVF, *in vitro* fertilization; RMB, renminbi. “Family history” denotes a self-reported relevant family history.

### 3.2 Thematic findings

**Figure 1:**
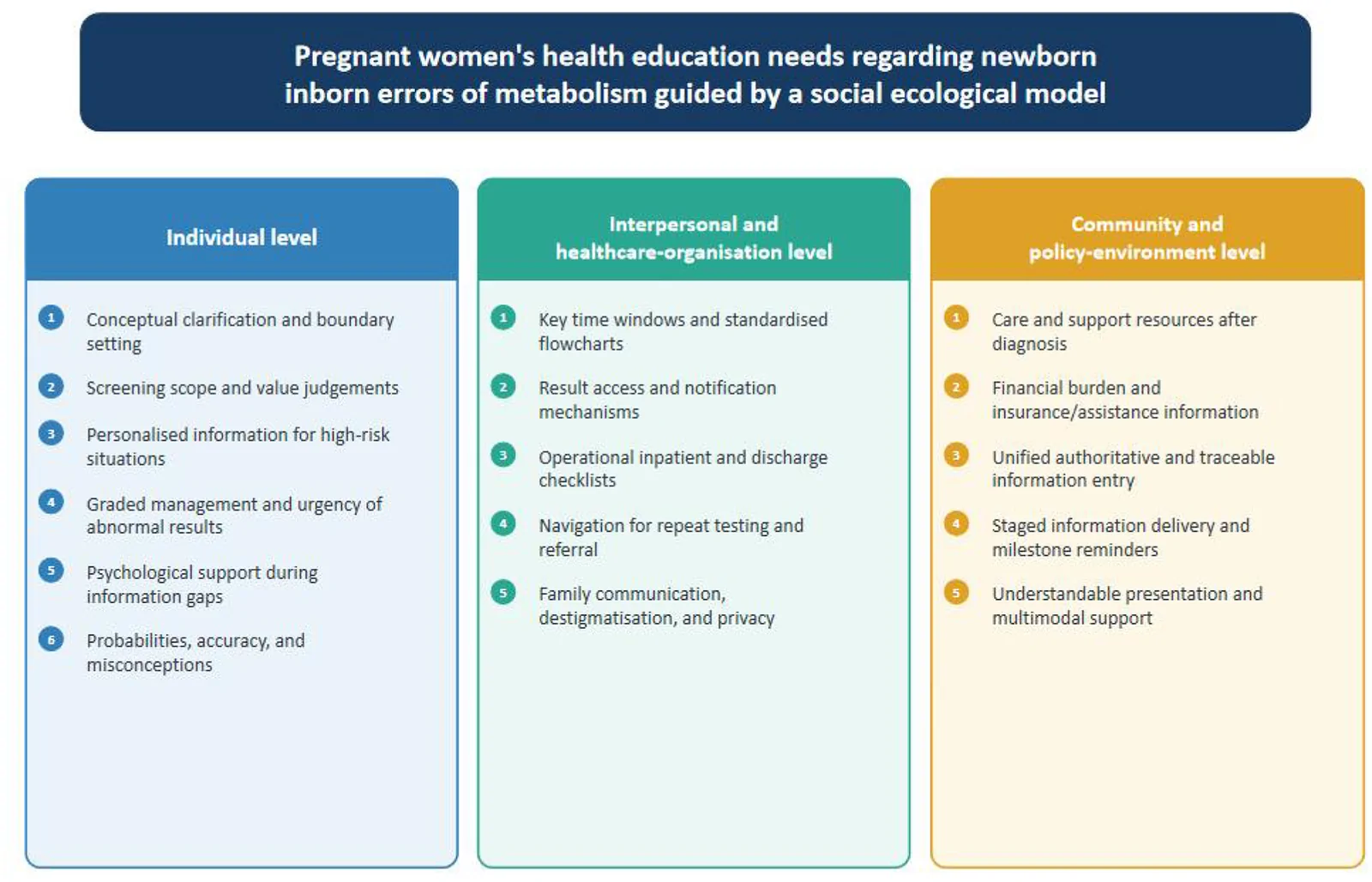
Three-level thematic structure of pregnant women's health education needs regarding newborn inborn errors of metabolism guided by a social ecological model.

Using Colaizzi's seven-step method, 16 inductively generated subthemes were organized into three reporting levels: individual; interpersonal and healthcare organization; and community and policy environment. The structure moves from understanding, risk appraisal, and actionable capability; to family/clinician communication and closed-loop service processes; and then to resource accessibility, affordability, and the wider information environment. The final three interviews (N13–N15) added no new subthemes. Figure 1 presents the three-level thematic structure, and Table 2 presents the levels, subthemes, codes, and contributing participant identifiers in the same order.

**Table 2:** Analytic levels, subthemes, and supporting codes for pregnant women's health education needs regarding newborn inborn errors of metabolism.

Analytic level	Subtheme	Codes and contributing participants
Individual level	Conceptual clarification and boundary setting	Unfamiliarity with the concept of newborn IEM (N3, N4, N6, N7, N12, N14, N15) Confusion between prenatal testing and newborn screening (N1, N2, N3, N5, N6, N9, N11) Understanding screening as risk indication rather than diagnosis (N2, N3, N5, N7, N12, N13)
	The scope of screening and judgements about its value	Need to know the scope of screening items (N1, N2, N3, N4, N5, N6, N7, N9, N11, N12, N14, N15) Perceived benefits, harms, and value of expanded screening (N5, N12)
	Personalized information needs for high-risk groups	Communication about genetically related risk and follow-up pathways (N1, N2, N5, N9, N10, N12, N13, N14, N15) Need for clear knowledge boundaries to reduce excessive worry (N5, N6, N9, N12) Need for personalized counseling for high-risk groups (N13)
	Graded management of abnormal results and judgement of urgency	Judging urgency and receiving graded management after abnormal results (N1, N2, N3, N4, N5, N7, N8, N11, N12, N13, N14) Symptom warning levels and advice on when to seek care (N2, N12) Scenario-based examples to improve understanding (N5)
	Anxiety during information gaps and the need for psychological support	Health education should be adapted to different literacy levels (N2, N3, N4, N9) Anxiety affects daily functioning (N1, N3, N4, N6, N10, N12, N15)
	Communicating probabilities, accuracy, and common misconceptions	Notification methods should accommodate lower health literacy (N1, N2, N3, N4, N5, N7, N12, N13, N14) Health education should emphasize probabilities and pathways (N2) Negative results still require rational interpretation (N2, N5, N7, N13) Clarification of common misconceptions (N12, N14)
Interpersonal and healthcare-organization level	Key time windows and standardized flowcharts	Need for key time windows and process timelines (N1, N2, N3, N4, N5, N6, N7, N9, N11, N12, N13, N14, N15) Concern about missing critical time windows (N1, N2, N3, N4, N7, N9, N10, N11, N12, N13)
	Result access and notification mechanisms	Unclear pathways for result checking (N3, N4, N7, N12, N13) Multichannel result notification and reminders (N7, N12, N15) Notification methods should suit different groups (N15)
	Operational checklists for inpatient and discharge settings	Information overload during hospital stay (N4, N6, N7, N8, N10, N11, N12, N14, N15) Information may be missed when handled by family members (N4) Postpartum fatigue affects information uptake (N7, N12)
	Navigation for repeat testing and referral	Repeat testing, referral, and procedural pathways after abnormal results (N1, N2, N3, N4, N5, N6, N7, N8, N9, N10, N11, N12, N13, N14, N15)
	Family communication, destigmatisation, and privacy concerns	Family-oriented materials and standard explanation scripts (N1, N2, N3, N4, N5, N7, N9, N10, N12, N13) Addressing traditional beliefs and resistance (N1, N3, N4, N5, N12, N15) Privacy protection and concerns about labeling (N2, N5, N9, N13) Non-blaming and destigmatising communication (N1, N2, N3, N4, N5, N6, N7, N8, N9, N12, N13, N14)
Community and policy-environment level	Care and support resources after diagnosis	Need for care and long-term management after diagnosis (N1, N2, N3, N4, N5, N6, N7, N8, N9, N10, N11, N12, N13, N14, N15) Providing hope and feasible pathways (N4, N6, N12) Transport and caregiving burden related to repeat testing (N4, N7, N10, N12)
	Financial burden and insurance/assistance information	Cost sensitivity and weighing value (N5, N6) Concern about long-term financial pressure (N1, N2, N3, N4, N5, N6, N7, N8, N9, N10, N11, N12, N13, N14, N15)
	A unified authoritative entry point and traceable information	Need for a unified authoritative information entry point (N3, N4, N7, N9) Closed loop of result checking, explanation, and next-step guidance (N7)
	Staged information delivery and reminders at key nodes	Need for layered presentation of information (N1, N2, N4, N5, N7, N9, N12, N14) Reminders at key milestones (N4, N5, N9, N11)
	Easy-to-understand presentation and multimodal support	Need for flowcharts plus action checklists (N1, N2, N3, N4, N5, N6, N7, N8, N9, N10, N11, N12, N13, N14) Need for flowcharts plus short videos (N2, N4, N7, N9, N11, N12, N14) Opposition to fear-based education, with emphasis on probabilities and pathways (N8, N11, N15) Prioritized 80/20 key information for busy populations (N2, N4, N5, N12) Priority for flowcharts and post-abnormal-result guidance, with FAQs and question lists as supplements (N1, N3, N4, N5, N11, N12, N13, N15)

IEM, inborn errors of metabolism. Participant identifiers (N1–N15) indicate the interviews contributing to each code. Subthemes are ordered identically in the Results, Table 2, and Figure 1.

#### 3.2.1 Individual-level health education needs

At the individual level, participants' needs centered on constructing a usable cognitive framework for IEM and NBS, understanding risk and uncertainty, and gaining the skills and emotional support needed to act on results.

- **Theme 1: conceptual clarification and boundary setting**

Most participants were familiar with heel-prick blood sampling after birth, but were unfamiliar with the term “inborn errors of metabolism.” They also tended to confuse prenatal testing with newborn screening and wanted a clear and transferable explanatory framework. “I know about newborn screening-that heel blood test after my first baby was born. But ‘inborn errors of metabolism'… I'm not really familiar with that term” (N3). “Prenatal tests check whether the fetus has problems, and the postnatal ones check whether the baby has a disease? But I can't tell which conditions can be detected during pregnancy and which have to wait until after birth” (N1).

- **Theme 2: the scope of screening and judgements about its value**

Participants wanted to know exactly which conditions are screened for, how severe they are, and whether they are treatable or controllable. They preferred a layered presentation with clear reasons to support value judgements. “First, what diseases are actually included in newborn screening? Which ones are severe, and which can be treated or controlled?” (N1). “It would be best if the information were stratified. I can't remember a very long list, but I do want to know the key points and why they matter” (N2). Some women also wanted an authoritative explanation of the advantages and disadvantages of expanded screening and the anxiety caused by false-positive results: “I'd prefer an authoritative explanation of whether more screening is really worth it and who it is suitable for” (N5).

- **Theme 3: personalized information for women in high-risk situations**

When there was a family history, assisted reproduction, or a previous adverse pregnancy outcome, participants emphasized the need for personalized information about what was relevant to them and where the actual risk boundaries lay, so as to reduce unnecessary worry. “I'm more concerned about how ‘genetic risk' is explained and what the follow-up pathway looks like” (N1). “I want someone to give me a boundary: what I don't need to worry about now, and what needs to be handled after birth according to the process” (N5). “For someone like me with a family history, if the screening is normal, can I really feel reassured?” (N13).

- **Theme 4: graded management of abnormal results and judgement of urgency**

Participants generally worried that if they received a notification of an “abnormal” or “elevated” result, they would not know how urgent it was. They wanted an explanation that “abnormal does not mean confirmed diagnosis,” together with graded management plans specifying timelines, locations, appointments, and required materials. “The nurse just said it was ‘a bit high' and that I should come back for repeat testing as soon as possible, but she didn't explain whether it was serious” (N4). “First, tell me how serious it is and how urgent it is; second, give me a clear next step; and third, tell me that abnormal does not equal diagnosis” (N1). Participants also wanted scenario-based examples and red-yellow-green warning cues to make the information more actionable: “If there were a red-yellow-green prompt sheet, I'd find that very practical” (N2). “I want to know how to interpret a ‘suspicious' text message and how to arrange the next step” (N5).

- **Theme 5: anxiety during information gaps and the need for psychological support**

During periods of waiting for explanation or repeat testing, participants often became anxious and turned to online searching. They felt that a message consisting only of “repeat testing recommended” amplified uncertainty and wanted timely reassurance together with clear guidance. “After an abnormal screening prompt comes out… if there's only one sentence saying ‘repeat testing recommended', with no explanation and no pathway… anxiety grows exponentially” (N2). “The more I read, the more confused I become, and then it even affects my sleep” (N1).

- **Theme 6: communicating probabilities, accuracy, and common misconceptions**

Participants felt that online information often “describes the consequences without discussing probability” and “states conclusions without explaining the premises.” They wanted probabilities explained through plain-language analogies, and misconceptions clarified, such as “screening does not equal diagnosis,” “a negative result does not mean complete exclusion,” and the meaning of false positives and false negatives. They also disliked fear-based health messages. “This kind of information often only talks about consequences and not probability, which makes people imagine the most extreme cases” (N1). “Probabilities should be explained using everyday analogies… Don't just present extreme negative cases; also include positive examples showing that some conditions are manageable and treatable” (N2).

#### 3.2.2 Interpersonal and healthcare-organization needs

At the interpersonal and healthcare-organization level, participants focused on interactions with family members and professionals and on the service processes connecting education, sampling, result notification, repeat testing, referral, and follow-up.

- **Theme 1: key time windows and standardized flowcharts**

Participants generally wanted a timeline and flowchart covering “blood sampling after birth → result release → result checking → abnormal/suspicious result → repeat testing/referral,” so that they would not miss important milestones. “What I need is: on which day after birth is the blood taken, how long until the result comes out, where can I check it, if it is abnormal or suspicious then what clinic should I register for, and by when must I go?” (N1). “A diagram plus a checklist would be best. The diagram gives the whole picture, and the checklist tells me what to do and what to bring” (N3).

- **Theme 2: result access and notification mechanisms**

Participants were uncertain about how to obtain results and how they would be notified. They expected multi-channel reminders and explanatory notifications that would clearly state where results could be checked, how abnormal results would be communicated, and whether they themselves needed to take the initiative if no result appeared. “At that time I didn't really know where to check the result… The nurse said it could be checked on the hospital's public account, but it still felt troublesome” (N3). “Ideally there should be dual reminders by phone plus text message or mini-program message, and the message should tell me what kind of situation it is, how urgent it is, and where to go next” (N7). Participants also highlighted the need to adapt notification methods for people with lower health literacy: “It would be best to call directly… I'm afraid I might not understand a text message” (N15).

- **Theme 3: operational checklists for inpatient and discharge settings**

Participants felt that the postpartum hospital stay involved a high information burden and fatigue, which interfered with comprehension and memory. They therefore suggested moving health education forward to the middle or late stages of pregnancy and providing materials that could be consulted repeatedly. “Don't wait until after delivery, when I'm in hospital, to tell me all this. There are too many things happening then, and I won't remember it” (N4). “After delivery I'll be exhausted… I'd much rather receive the information in advance and be able to look at it again later” (N7). They also noted that when family members handled procedures on their behalf, information could easily be lost: “Family members may not understand it either, so it's easy for information to be missed” (N4).

- **Theme 4: navigation for repeat testing and referral**

After an abnormal result, participants' most urgent questions were where to go, which department to attend, how to make an appointment, and what to bring. They hoped for a combination of automated reminders and a clearly identified consultation contact to ensure a closed loop. “First, I can't understand the technical terms-metabolic pathways, enzyme deficiencies and so on just make me blank; second, there's no clear process, so I don't know where to go, which specialty to see, or how soon I must go; third, there's the emotional side-once I get more anxious, I just want to avoid it” (N1). “The system should automatically push key reminders: the sampling time, the time for checking results, and the deadline for repeat testing. At the same time there should be a clear consultation window, such as a genetic counseling clinic or a neonatal follow-up nurse's phone number” (N2).

- **Theme 5: family communication, destigmatisation, and privacy concerns**

Participants pointed out that differences in how family members interpreted “abnormal” results and probabilities could lead either to denial or to excessive panic, affecting shared decision-making. They were also concerned about the stigmatizing label of “genetic disease” and about privacy breaches, and they hoped for family-facing educational materials and standard explanation scripts. “We need a script for family members so that they know the next step is not a ‘verdict', but rather ‘following the process to rule things out”' (N1). “Older family members worry that even talking about it is inauspicious” (N3). “Many family members think ‘abnormal equals disease'… and there are also privacy issues, because people worry about being labeled” (N2).

#### 3.2.3 Community and policy-environment needs

At the community and policy-environment level, participants' needs concerned the availability and affordability of support, access to trustworthy information, privacy and stigma, and institutional mechanisms that reduce the practical burden of acting on screening results.

- **Theme 1: care and support resources after diagnosis**

Participants wanted access to long-term management pathways in the event of a confirmed diagnosis, including follow-up, diet or caregiving points, and support resources. Knowing that “there is still a path forward” was seen as important for buffering fear and helplessness. “If the diagnosis were really confirmed, I'd want to know how to care for the child day to day-diet, follow-up, and whether there are support resources” (N1). “At the very least, I need to know that once it's diagnosed, it's not that the sky is falling-there is still a way forward” (N4).

- **Theme 2: financial burden and insurance/assistance information**

Participants wanted to know in advance the likely costs of screening, repeat testing, and further examinations, as well as reimbursement policies, so that families could plan budgets and weigh value. “I want to have a rough idea: how much basic screening costs, how much repeat testing might cost, and whether medical insurance can reimburse it” (N1). “If it's the kind of test that costs several thousand yuan, I'd hesitate about whether it's worth it” (N5). Some participants also voiced concerns about long-term financial pressure: “What worries me most is whether our family could afford it if a problem were found” (N1).

- **Theme 3: a unified authoritative entry point and traceable information**

Participants felt that online information was fragmented and could easily provoke panic. They preferred healthcare institutions to provide a single, traceable information source that integrated result checking, explanation, and guidance for the next step. “Ideally there should be one fixed entry point… telling me when to do what, how to check the result, and what to do if the result is abnormal” (N3). “It would be best if the whole chain-check the result, read the explanation, and see the next-step guidance-were integrated” (N7).

- **Theme 4: staged information delivery and reminders at key nodes**

Participants generally supported staged information pushes matched to pregnancy stages and reminders at key milestones, stressing that it was not a matter of “more is better,” but rather of information appearing exactly when needed. “In the second and third trimesters, first give the basic concepts; in late pregnancy, provide the process checklist; remind us once again during the hospital stay… and after discharge send information on how to check the results” (N1). “The key isn't having more information; it's that the information appears right when it's needed” (N2).

- **Theme 5: easy-to-understand presentation and multimodal support**

Participants preferred low-burden formats such as short videos, flowcharts, one-page summaries, and FAQs, and emphasized the need to avoid fear-based communication while taking the needs of people with lower health literacy into account. “I prefer short videos or a one-page summary of the key points. A flowchart would be even better” (N1). “If there's too much text, I can't take it in, and if the videos sound frightening, I won't dare watch them” (N8). Busy women, in particular, wanted an 80/20-style focus on the most important information: “What I need more is the 80/20 key points” (N2).

### 3.3 Theme saturation

The final three interviews (N13, N14, and N15) were used prospectively to test thematic saturation. They elaborated existing codes but generated no new subthemes. The subsequent mapping of inductive themes to the three social ecological reporting levels did not change the source codes or the saturation judgement.

## 4 Discussion

This study identified a linked sequence of health education needs extending from individual understanding to family and service coordination and then to structural access. The principal finding was not simply that pregnant women lacked knowledge. Rather, women wanted information that specified boundaries, urgency, and the next action at each transition from pregnancy to newborn screening and from an abnormal screen to repeat testing, diagnosis, and longer-term support. The three reporting levels were retained consistently across the Methods, Results, Table 2, Figure 1, and Discussion. They represent a transparent consolidation of McLeroy's five-level model (27), not Bronfenbrenner's original definition of a mesosystem.

At the individual level, the findings extend previous work in two ways. Earlier surveys and educational studies have mainly measured awareness, knowledge, attitudes, or preferences for antenatal information (14, 16–18). The present interviews additionally showed that women needed conceptual boundaries-particularly the distinctions between prenatal testing, NBS, repeat testing, and diagnosis-and probability explanations that prevented both false reassurance and catastrophic interpretation. This is important because expanding biochemical and genomic screening increases the amount and complexity of information that families must appraise (35–38). The desire for red-yellow-green urgency cues, scenario-based explanations, and explicit wording that “screen positive does not equal diagnosis” translates general health-literacy principles into concrete design requirements. Education should therefore be layered, use plain language and numerical or visual risk explanations, and pair every risk message with an actionable next step.

The individual-level accounts also make psychological support part of education rather than an optional addition. Existing studies have documented distress following false-positive or inconclusive results (19–23, 39–41), whereas participants in this study described the information gap itself as a trigger for repetitive searching, sleep disturbance, and avoidance. This distinction suggests that communication timing and pathway clarity are modifiable components of psychosocial care. A structured abnormal-result message should state what the result does and does not mean, the degree of urgency, the repeat-testing deadline, where to attend, whom to contact, and when urgent care is necessary. Such communication should be adapted for different health-literacy levels and should avoid fear-based framing.

At the interpersonal and healthcare-organization level, the findings move beyond recommending greater family involvement or more professional education. Participants described a specific closed loop: anticipatory education during pregnancy, a sampling and result-access timeline, multi-channel notification, an inpatient/discharge checklist, repeat-testing and referral navigation, and confirmation that follow-up had occurred. Prior research has shown that antenatal information is often inconsistently delivered and that communication after abnormal results may be poorly understood, especially by people with limited health literacy (18, 24, 42–44). Our study adds the need for family-facing explanation scripts that reduce blame, stigma, and conflict while protecting privacy. The findings support embedding brief, repeatable NBS education in routine antenatal care and designating responsibility for each transition rather than relying on a single postpartum information encounter.

The emphasis on value judgements and uncertainty also differs from purely informational approaches. Pregnant women wanted to understand not only which disorders were included but why expanded screening might be useful, what false-positive or uncertain findings could mean, and which choices were sensitive to family values. Recent qualitative work with expectant parents considering genetic NBS similarly identified concerns about certainty, psychological consequences, and social implications (45). Decision-support strategies that combine balanced education with values clarification may therefore be appropriate for optional expanded or genomic screening (46, 47). However, decision aids should complement rather than replace professional counseling and a reliable follow-up pathway.

At the community and policy-environment level, participants linked information needs to affordability, travel and caregiving burdens, privacy, and the availability of long-term management resources. These concerns were especially salient across the sample's wide range of education, residence, parity, and family income. Health-literate systems require responsive organizations, a prepared workforce, people-centered services, trustworthy technology, and supportive financing rather than placing the full burden on individuals (48). A unified institutional entry point should integrate result checking, plain-language interpretation, contact details, referral navigation, and information on insurance or assistance. Digital reminders and short videos may extend reach, but they require offline alternatives, privacy safeguards, and clinician back-up (49–52).

## 5 Conclusions

This descriptive phenomenological study identified 16 subthemes across three consistently applied social ecological levels. Individual-level needs concerned understandable risk information, emotional support, and actionable skills. Interpersonal and healthcare-organization needs concerned shared family understanding and a closed service loop from antenatal education through notification, repeat testing, referral, and follow-up. Community and policy-environment needs concerned authoritative information, affordability, privacy, and access to continuing care and support.

The findings support a staged intervention beginning in pregnancy and extending beyond birth: foundational concepts and screening choices during antenatal care; practical timelines and checklists before delivery; clear result-access instructions around discharge; and graded, supportive navigation after abnormal results. Evaluation should assess not only knowledge but also comprehension, decisional conflict, anxiety, follow-up timeliness, referral completion, and equity of access across health-literacy and socioeconomic groups.

This study has strengths and limitations. Purposive variation across trimester, conception mode, parity, education, urban/rural residence, income, and relevant reproductive or family history increased information power and transferability (53). Independent coding, third-researcher adjudication, transcript return, reflexive documentation, and saturation testing strengthened analytic credibility. Nevertheless, all 15 participants were recruited from one institution, so the findings may not transfer to regions with different screening panels, financing arrangements, or referral systems. The sample included socioeconomic and educational diversity, but it was not designed for subgroup comparison, and previous personal experience of NBS was not systematically recorded as a separate characteristic.

## Data Availability

The raw data supporting the conclusions of this article will be made available by the authors, without undue reservation.
